# The mineralocorticoid receptor knockout in medaka is further validated by glucocorticoid receptor compensation

**DOI:** 10.1038/sdata.2017.189

**Published:** 2017-12-12

**Authors:** Tatsuya Sakamoto, Madoka Yoshiki, Hirotaka Sakamoto

**Affiliations:** 1 Ushimado Marine Institute, Faculty of Science, Okayama University, Setouchi 701-4303, Japan

**Keywords:** Homeostasis, Animal physiology

## Abstract

To study the critical role of mineralocorticoid signalling, we generated a constitutive mineralocorticoid receptor (MR)-knockout (KO) medaka as the first adult-viable MR-KO animal. This KO medaka displayed abnormal behaviours affected by visual stimuli. In contrast, the loss of MR did not result in overt phenotypic changes in osmoregulation, despite the well-known osmoregulatory functions of MR in mammals. Since glucocorticoid receptor (GR) has been suggested to compensate for loss of MR, we examined expression of duplicated GRs with markedly different ligand sensitivities, in various tissues. qRT-PCR results revealed that the absence of MR induced GR1 in the brain and eyes, but not in osmoregulatory organs. This reinforces the important functions of glucocorticoid signalling, but the minor role of mineralocorticoid signalling, in fish osmoregulation. Because both 11-deoxycorticosterone (DOC) and cortisol are ligands for MR, whereas GRs are specific to cortisol, GR1 signalling may compensate for the absence of cortisol-MR, rather than that of DOC-MR. Thus, this GR expression suggests that our MR-KO model can be used specifically to characterize DOC-MR signalling.

## Background & Summary

Gene targeting techniques are powerful tools for elucidating the function of specific genes. However, the studies are often complicated when the absence of a gene results in lethality or exerts pleiotropic effects, making it difficult to distinguish between direct and indirect actions. This is especially true in the study of corticosteroid systems, which are involved in two major functions in vertebrates: a glucocorticoid function that affects metabolism and growth, and a mineralocorticoid function that regulates transport of ions and water. These functions are associated with two hormones, cortisol (or corticosterone) and aldosterone, which activate the glucocorticoid receptor (GR) and mineralocorticoid receptor (MR); however, these receptors have been reported to compensate for the loss of each other.

In teleost fish, it has long been held that a single hormone, cortisol, has both glucocorticoid and mineralocorticoid actions^1^, since fish lack aldosterone^2,3^. The counterparts of tetrapod MRs and their specific endogenous ligand, 11-deoxycorticosterone (DOC), have only recently been identified^4–6^. Furthermore, whole genome duplication in the teleost lineage has given rise to 2 GR isoforms that show marked differences in ligand sensitivity in several species, including medaka^7^. The effects of MR/GR agonists/antagonists and the dynamics of expression of cortisol/DOC-MR/GRs indicate a major role of cortisol-GRs not only in ‘glucocorticoid function’ but also in osmoregulation, compared with that of mineralocorticoid signalling (DOC/cortisol-MR)^8^. However, neither the function of the fish mineralocorticoid system nor the distinct role of the two GRs has been defined.

In our coupled, main paper published in Nature’s *Scientific Reports*, we have very recently generated a constitutive MR-knockout (KO) medaka as the first adult-viable MR-KO animal, and shown that MR is required for behaviours affected by visual stimuli, but is not essential for osmoregulation in teleost fish^9^. These results may reveal a phylogenetically conserved link between mineralocorticoid signalling and behaviour, since this signalling is implicated in control of brain-behaviour actions, in addition to the osmoregulatory role, in higher vertebrates^10^.

To validate these conclusions, it is critical to examine the effects of MR loss on compensation by other members of the corticosteroid receptor family. However, targeted disruption of MR in mice results in neonatal lethality, and thus precludes analysis of these receptors in many adult tissues^11,12^. The studies presented herein have circumvented this problem by examining expression of GR in various tissues using the above MR-KO medaka.

**Figure 1:**
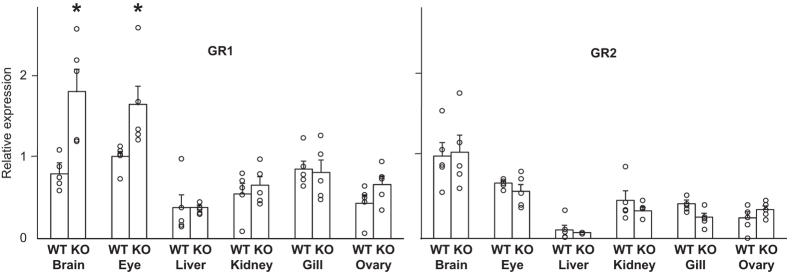
GR1, but not GR2, is upregulated specifically in brain and eyes of MR-KO medaka. Expression of GRs in tissues of MR-KO and WT adult (200 days post-fertilization) medaka was determined by qPCR. Each fish was analyzed in triplicate. mRNA levels are normalized (see Methods; raw qRT-PCR data are provided in Data Citation 1) and shown as fold differences from maximum mean levels in WT. Mean±SEM (*n*=5 in each group) are also shown. *P<0.05.

qRT-PCR revealed that both GR isoforms are expressed ubiquitously in various tissues of wild-type (WT) adult medaka (Fig. 1), as opposed to MR expression, which is modest in osmoregulatory organs (kidney and gill) but high in the brain, as for most teleosts^8,9^. In MR-KO fish, although osmoregulation was normal, expression of both GRs in osmoregulatory organs was similar to that in WT. The loss of MR in the osmoregulatory organs does not appear to be compensated for by induction of GRs. These results reinforce the important functions of glucocorticoid signalling in fish osmoregulation and the minor role for mineralocorticoid signalling^8,9^. On the other hand, MR loss induced expression of GR1, but not GR2, in the brain and eyes. Thus, the ability of GR to compensate for MR loss may be dependent on the tissue and/or gene targets. Because both DOC and cortisol are ligands for MR, whereas GRs are specific to cortisol, the central GR1 induction may suggest that GR1 signalling may specifically compensate for the absence of cortisol-MR, which provides the first convincing evidence for a distinct role of GR1, and that DOC-MR is not subject to compensation by GRs. However, the amounts of ligands are also important to confirm this signalling system compensation. Regardless, disruption of DOC-MR might be responsible for our MR-KO phenotype (Table 1).

Ideally, studies performed using MR/GR1/GR2 double/triple null animals are required to establish the role of corticosteroid receptors definitively, but these animals will probably not survive to adulthood. Mouse lines with conditionally altered expression or function of corticosteroid receptors^13^ as an alternative strategy have incomplete or complex effects. However, the present analysis suggests that our MR-KO model will permit further characterization of ‘mineralocorticoid’ (DOC-MR) signalling, rather than that of cortisol-MR. This model also provides an opportunity for identifying the physiologically distinct roles of two GRs, which are not defined partly due to the complicated interaction with mineralocorticoid signalling.

## Methods

### Fish

#### Ethics

The handling, care and use of the Hd-rR strain medaka (*Oryzias latipes*) in this study were carried out using standard procedures^14^ in accordance with the Animal Research Guidelines at Okayama University. All procedures were approved by the Committee for Animal Research, Okayama University. All efforts were made to minimize suffering by careful housing and husbandry. Animal welfare checks were performed when the lights came on and before leaving at the end of the day for all fish by visual inspection to assess abnormal appearance^9^. Before handling, fish were rapidly anaesthetized with 0.01% tricaine methane sulfonate (Sigma) neutralized with sodium bicarbonate.

#### Study design and housing/husbandry conditions

The strains were raised to adulthood and reared in colony tanks connected to the recirculating system at 27 ± 2 °C with a 14:10 h light:dark cycle. The MR-KO mutant is described in our coupled paper in *Scientific Reports*^9^ to exhibit no signs of physical abnormalities and was genotyped using the primer pair: forward 5′- TGT CCA GCC CTC ACA GTA TG-3′; reverse 5′- GGC TGC TGC TAT CGT TCT G-3′^9^. The random allocation of fish to experimental group (WT versus MR KO) was driven by Mendelian inheritance. The experimental unit was considered the individual fish. Adult fish (*n*=5) were randomly selected and were placed in aged tap water of plastic static containers measuring 410×250×200 (L×W×D) (volume of water 6000 mL) held on a rack in a room with a controlled environment of a temperature of 27±2 °C and a 14:10 h light:dark cycle. Singly housed group (WT or MR KO) of fish were fed twice per day with a powdered diet (Tetra-fin, Tetra Werke Co., Melle, Germany). Water was changed every 2 days to maintain the water quality. In the early afternoon (6–9 h after light onset; 1300–1600 h) after 5 days, brain, eyes, liver, kidney, gill and ovary were removed, with no specific order in which the fish in the two groups were processed. Researchers were not blinded to genotype, but they had no *a priori* knowledge or assumption as to the experimental outcome.

### Gene Expression of *gr1, gr2 and mr* by qRT-PCR

Total RNA from brain, eyes, liver, kidney, gill and ovary of MR-KO and WT adult was extracted using an RNeasy Plus mini kit (Qiagen). RNA quantity was determined by a Qubit Fluorometer (Invitrogen). Total RNA was reverse transcribed using an Omniscript RT Kit (Qiagen). qRT-PCR was performed using an ABI Prism 7000 (Applied Biosystems) or MiniOpticon™ Real-Time PCR Detection System (Bio-Rad) and SYBR Premix Ex Taq II (Takara)^9,15–17^. Standard operating conditions were 95 °C, 5 min, followed by 40 cycles at 95 °C for 11 s and 60 °C for 90 s. In each experiment, housekeeping genes RPL-7 (*rpl7*) were quantified concurrently. Gene-specific primers were designed [*gr1*: 5′- GCG AGA TAA GAC CCG AAG CA-3′ and 5′- GCC TTT AGT TCC ACC TTG TCC A-3′; *gr2*: 5′- GCA CGA TGC TTT CCT GTC C-3′ and 5′- CGG CAT TAC TCT CAG CCA CA-3′; *mr*: 5′- CCA GAG GTG AAG GGT ATC CA-3′ and 5′- GAA GCC TCG TCT CCA CAA AC-3′; for *rpl7*^15,17^]. Results for *gr* and *mr* were normalized using *rpl7* mRNA levels.

### Statistics

Statistical analysis was carried out with Statview 5.0 (Abacus Concepts). Nonparametric Mann-Whitney U tests were used to compare means between MR-KO and WT for each organ.

## Data Records

The MR-KO strain has been deposited at the National BioResource Project (NBRP) Medaka as Strain ID MT1050 with descriptions (https://shigen.nig.ac.jp/medaka/strainDetailAction.do?strainId=11562). Raw qRT-PCR data for mRNA levels of *grs*, *mr* and *rpl7* with descriptions are provided in figshare (Data Citation 1). *mr* expression data with descriptions are given in our coupled manuscript^9^.

## Technical Validation

### Genotype Analysis by RFLP

Genomic DNA from tail fin biopsies was prepared and the region containing the target site of the TALEN was amplified, as described above. The resulting PCR products were digested at 37 °C for 30 min in 20 μL of DdeI restriction digestion solution that consisted of reaction buffer and 2 units of DdeI (New England Biolabs). The 264 bp PCR product was cut to yield 126 bp and 138 bp fragments for only WT alleles. These digestion products were analysed using agarose gels^9^.

### Quality control of qRT-PCR data

The dissociation curves of the primer pairs showed a single peak. Relative standard curves were constructed for *gr1*, *gr2, mr* and *rpl7* using cDNA stock from medaka RNA^9,15–17^. The *rpl7* mRNA level was relatively constant (Data Citation 1), as also shown previously^9,15–18^.

## Additional information

**How to cite this article:** Sakamoto, T. *et al.* The mineralocorticoid receptor knockout in medaka is further validated by glucocorticoid receptor compensation. *Sci. Data* 4:170189 doi: 10.1038/sdata.2017.189 (2017).

**Publisher’s note:** Springer Nature remains neutral with regard to jurisdictional claims in published maps and institutional affiliations.

## Supplementary Material



## Figures and Tables

**Table 1:** Possible roles of corticosteroid systems in medaka shown in our studies.

**Corticosteroid**	**Receptor**	**Brain-behaviour**	**Osmoregulation**
DOC	MR	Integration with visual responses	-
Cortisol		+	
	GR1	+	+
	GR2	+	
+, possible roles are shown; -, not possible. The details are described in our related manuscripts^8,9^. Central cortisol-MR and GR1 signalling may share the brain-behaviour functions.			

## References

[d1] figshare Sakamoto T. 2017 https://doi.org/10.6084/m9.figshare.c.3681778

